# Supplementary material for: Changes in the Habitat Preference of Crested Ibis (Nipponia nippon) during a Period of Rapid Population Increase
Source: Animals (Basel). 2021 Sep 7;11(9):2626. doi: 10.3390/ani11092626 (PMC8465559; doi:10.3390/ani11092626)
Supplement: Supplementary file 1 [file animals-11-02626-s001.zip › animals-1336102-supplementary.pdf]

Figure S1: The original rice paddies (green areas) and waterbodies (blue areas) in the upper panel, the predicted rice paddy index in the middle penal, and the predicted waterbody index in the lower panel. The rice paddy waterbody indices are predicted values of probability of presence of the wetland by random forest using longitude and latitude as explanatory variables. The red triangles are nest sites in 2019.

Figure S2: The values of the five important variables and wetland index (the product of rice paddy and waterbody) for nest site selection of the crested ibis at nests (circles) from 1981 to 2019, and the mean value of the variables at 900 pseudo-absence sites (horizontal lines).

Figure S3: The effect of rice paddy on the nest site selection of the crested ibis from 2000 to 2019.

Figure S4: The effect of waterbody on the nest site selection of the crested ibis from 2000 to 2019.

Figure S5: The effect of solar radiation in January on the nest site selection of the crested ibis from 2000 to 2019.

Figure S6: The effect of precipitation of wettest quarter on the nest site selection of the crested ibis from 2000 to 2019.

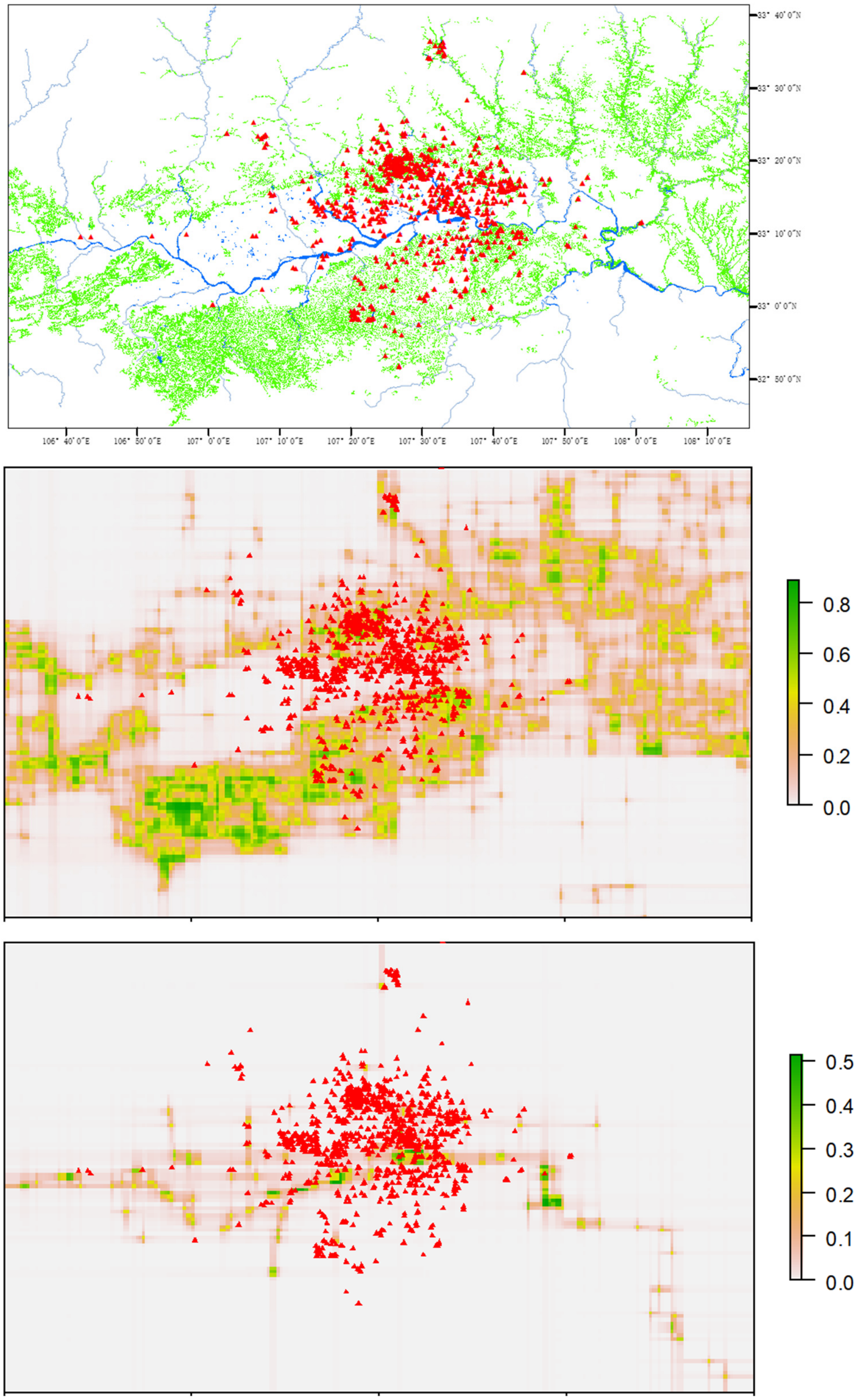

Supplementary Figure 1. The original rice paddies (green areas) and waterbodies (blue areas) in the upper panel, the predicted rice paddy index in the middle penal, and the predicted waterbody index in the lower panel. The rice paddy waterbody indices are predicted values of probability of presence of the wetland by random forest using longitude and latitude as explanatory variables. The red triangles are nest sites in 2019.

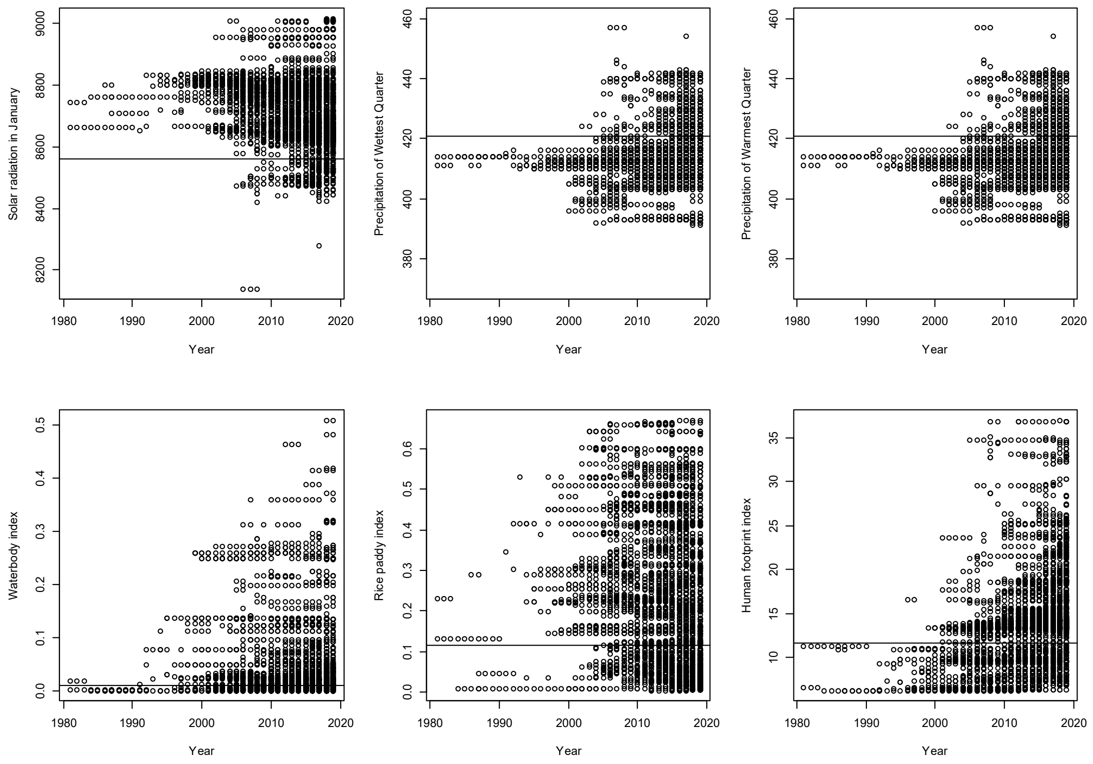

Supplementary Figure 2. The values of the five important variables and wetland index (the product of rice paddy and waterbody) for nest site selection of the crested ibis at nests (circles) from 1981 to 2019, and the mean value of the variables at 900 pseudo-absence sites (horizontal lines).

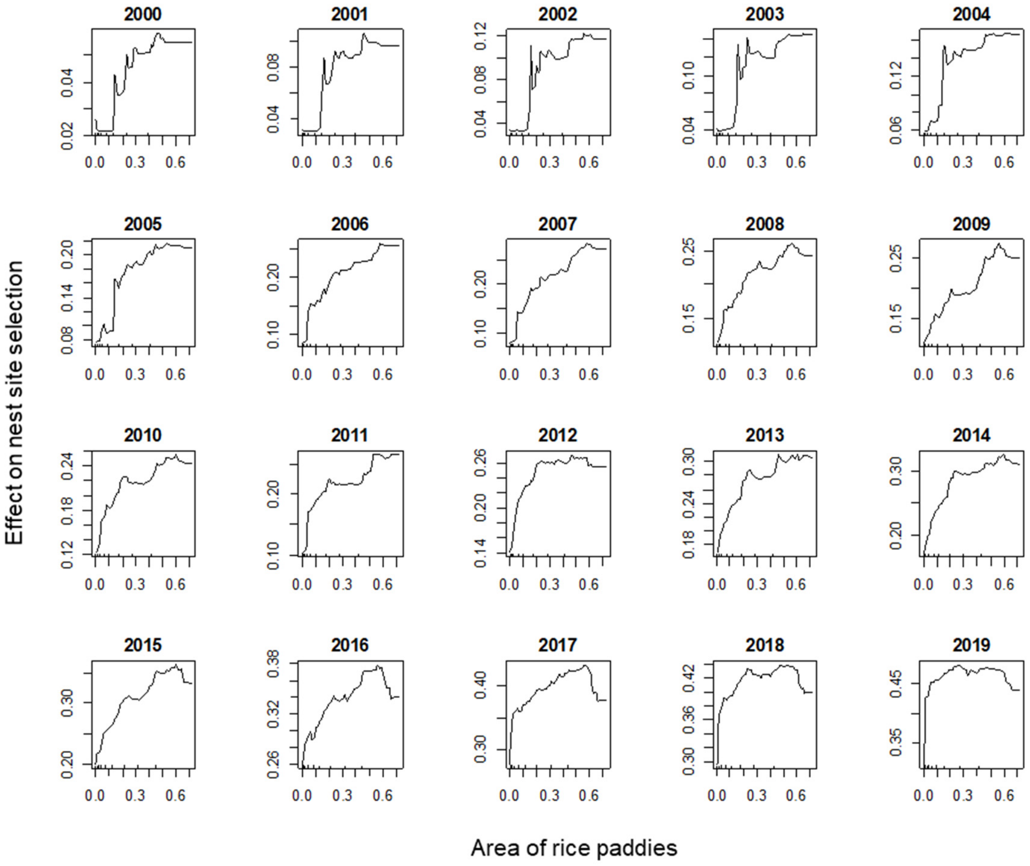

Supplementary Figure 3. The effect of rice paddy on the nest site selection of the crested ibis from 2000 to 2019.

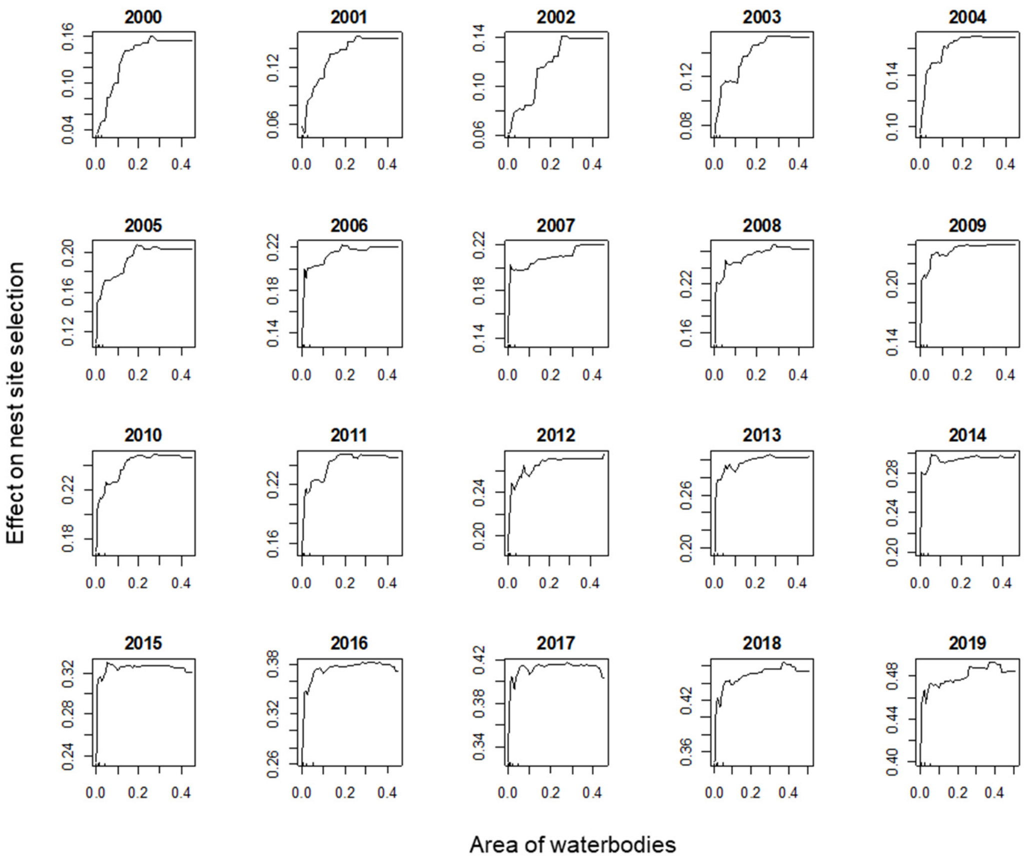

Supplementary Figure 4. The effect of waterbody on the nest site selection of the crested ibis from 2000 to 2019.

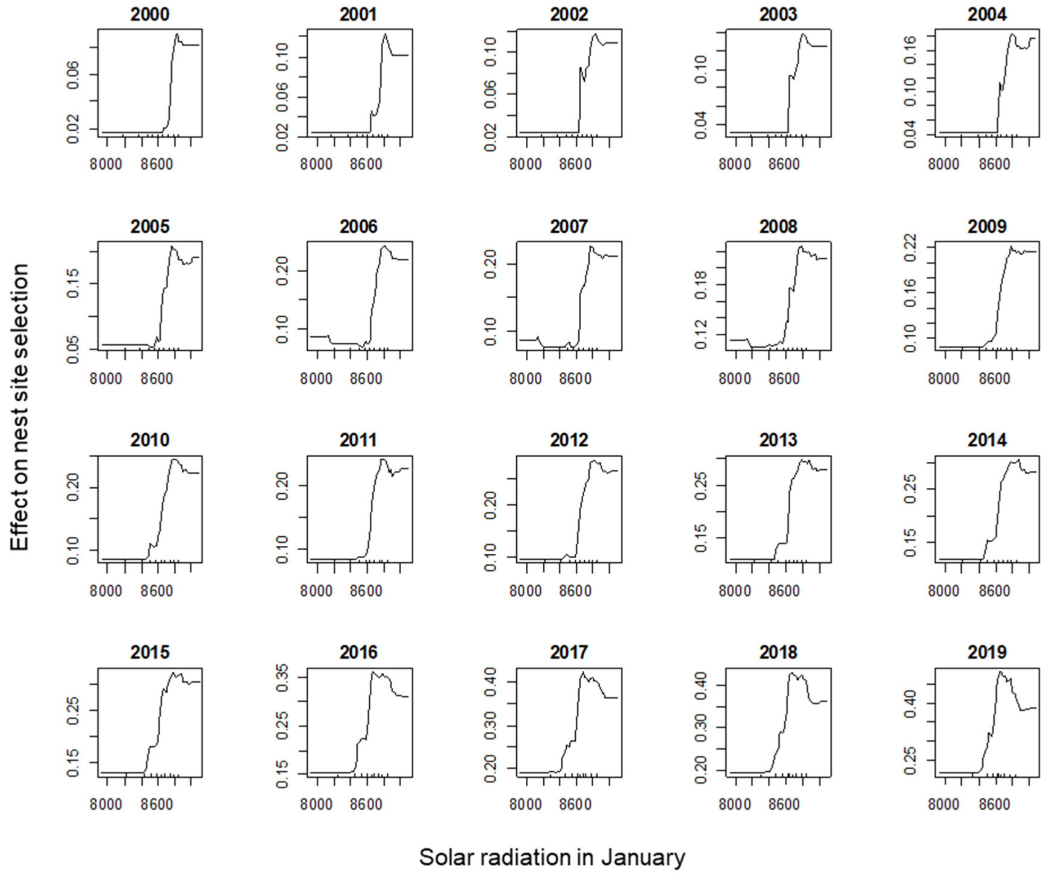

Supplementary Figure 5. The effect of solar radiation in January on the nest site selection of the crested ibis from 2000 to 2019.

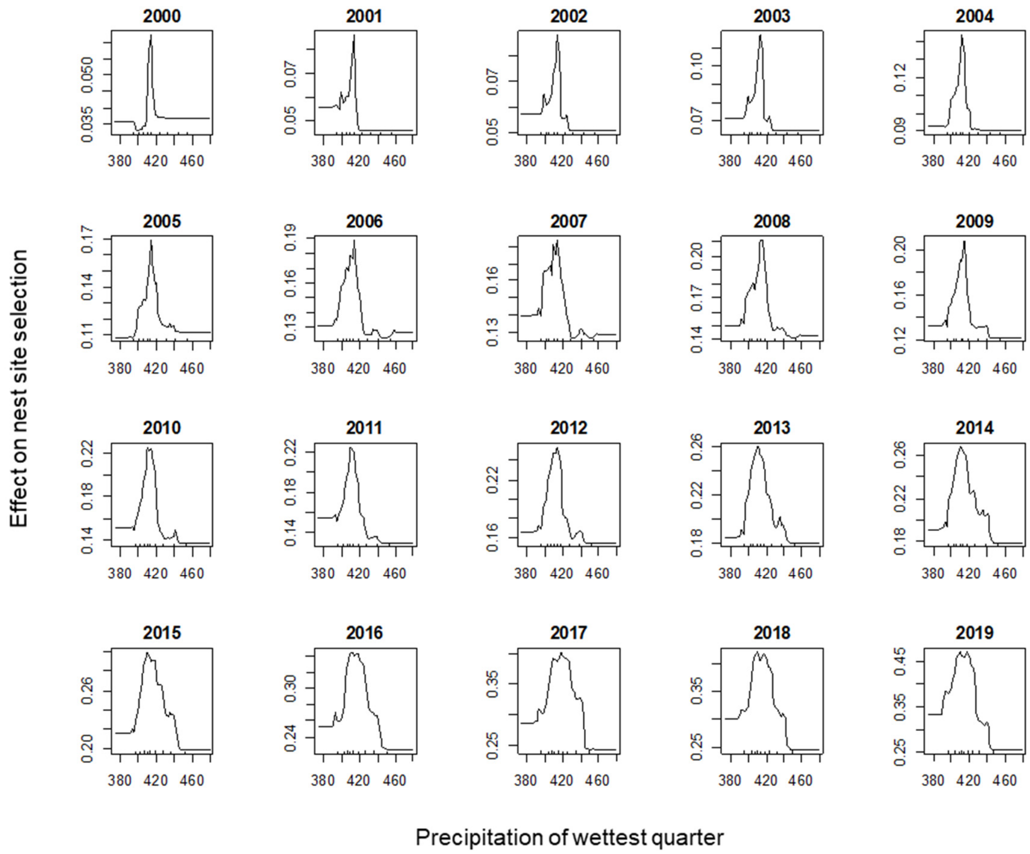

Supplementary Figure 6. The effect of precipitation of wettest quarter on the nest site selection of the crested ibis from 2000 to 2019.
